# Examining eating disorder symptoms, sociocultural pressures, romantic attachment, social support, mating approaches and mating tactics within romantic relationships

**DOI:** 10.1186/2050-2974-1-S1-O30

**Published:** 2013-11-14

**Authors:** Cassandra Dean, Janice Sabura Allen, Elizabeth Hughes

**Affiliations:** 1 School of Psychiatry and Psychology, Monash University, Australia; 2 Centre for Adolescent Health, The Royal Children's Hospital, Australia

## 

The current study examined the associations between romantic relationship status, gender, and factors of wellbeing related to eating disorders. A community sample of 588 women and 208 men, including a subsample of 80 couples, completed a series of self-report questionnaires. Involvement in such a relationship was related to less eating disorder symptoms, less attachment anxiety and avoidance, and greater social support, but was not related to the experience of sociocultural pressures. A more committed sexual approach style and more intimate mating tactics were found for those involved in a relationship compared with single status individuals. Within couples, partners were similar with regard to their level of perceived social support, global sociosexuality and use of friendship mating tactics. Women reported higher eating disorder symptoms, more sociocultural pressures and greater anxious attachment than men. The examination of relationship status and wellbeing, particularly eating disorder symptoms, is unique. The findings supplement the eating disorder literature and enhance the knowledge of mating behavior.

This abstract was presented in the **Disordered Eating – Characteristics & Treatment** stream of the 2013 ANZAED Conference.

